# A further study on *Franciscobasis* Machado & Bedê, 2016 (Odonata: Coenagrionidae), a newly described genus from Minas Gerais, Brazil

**DOI:** 10.1371/journal.pone.0223241

**Published:** 2019-10-08

**Authors:** Diogo Silva Vilela, Ricardo Koroiva, Adolfo Cordero-Rivera, Rhainer Guillermo-Ferreira

**Affiliations:** 1 Graduate Program in Entomology, Department of Biology, University of São Paulo (USP), Ribeirão Preto, Brazil; 2 Laboratory of Ecological Studies on Ethology and Evolution (LESTES), Department of Hydrobiology, Federal University of São Carlos, São Carlos, Brazil; 3 Coordenação de Biodiversidade, Instituto Nacional de Pesquisas da Amazônia, Manaus, Amazonas, Brazil; 4 ECOEVO Lab, Departamento de Ecoloxía e Bioloxía Animal, Universidade de Vigo, Pontevedra, Spain; Nanjing Agricultural University, CHINA

## Abstract

The genus *Franciscobasis* Machado & Bedê, 2016 is endemic to the Serra da Canastra National Park in Minas Gerais state, Brazil. Two species of *Franciscobasis* were described simultaneously with the genus description: *F*. *franciscoi* and *F*. *sonia*, the latter described only from females. Through morphological and molecular analysis, we investigated if *F*. *sonia* may represent the young female of *F*. *franciscoi*. Resulting data did not present adequate differences between females to characterize them as different species. Therefore, we suggest that *F*. *sonia* is a junior synonym of *F*. *franciscoi*, and the female of *F*. *franciscoi* goes through a complex ontogenetic color change.

## Introduction

*Franciscobasis* Machado & Bedê comprises a small Coenagrionid genus, which was described in 2015 to include two species: *F*. *franciscoi* and *F*. *sonia*, collected in the headsprings of the São Francisco River. On that occasion, seven other species were reported as new (five of them described in the same study); all of them were found in a 600 m transect, within Serra da Canastra National Park (SCNP, [1]). *Franciscobasis sonia* was an exception among the newly found species, having only females described, with males unknown. Furthermore, the separation between females of *F*. *sonia* and *F*. *franciscoi* was based mainly on coloration [1].

Delimitating species based solely on morphological methods may result in identification mistakes which can lead to the description of new species [2], especially in polymorphic taxa, which are very common in the family Coenagrionidae. Therefore, several authors advocate for the urgent need to apply integrative approaches to taxonomy and systematics [3]. Integrating different approaches and disciplines (such as molecular, external and internal morphology, and animal coloration) may provide useful data for biological reasoning behind descriptions of diverse life [4,5].

In order to further discover new species, the still unknown females of some species, and the male of *F*. *sonia*, we conducted five expeditions to SCNP, where we found a well-established population of *Franciscobasis*. The intriguing fact is that, despite finding males of *F*. *franciscoi* and females of both species, we could not find the male of *F*. *sonia*. Thus, faced with: (i) The great morphological resemblance of both females, (ii) the fact that no *F*. *sonia* males were found, (iii) the common occurrence of female polymorphism cases in Coenagrionidae [6–13], and (iv) the evidence that females can go through a complete coloration change over their ontogenetic development [9,14,15], we studied *F*. *franciscoi* and *F*. *sonia*, integrating molecular and morphological methods, hypothesizing that *F*. *sonia* might be an orange color morph of *F*. *franciscoi*, or even be a young female yet to complete its ontogenetic development.

## Materials and methods

### Collection and identification of specimens

**Fig 1:**
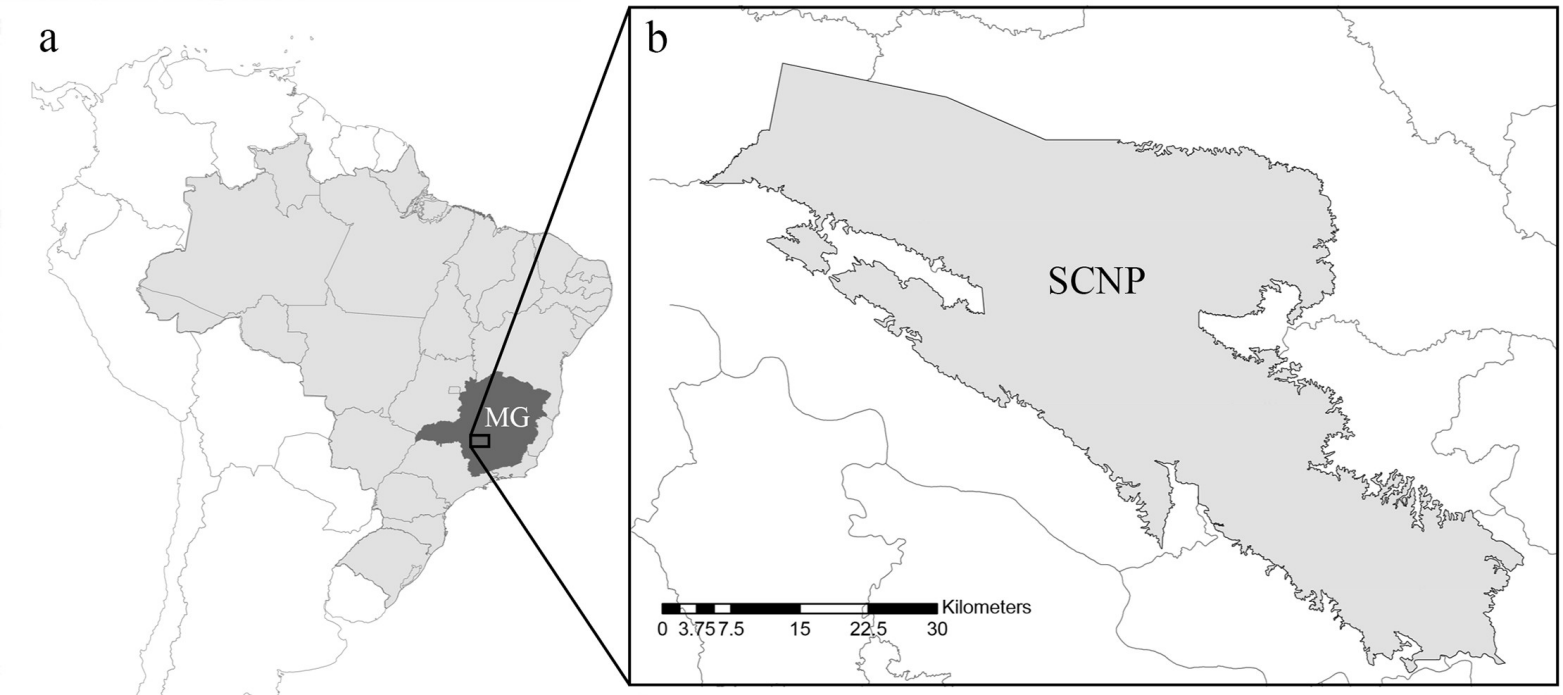
Map showing the locality of occurrence of *Franciscobasis sonia* and *F*. *franciscoi*. a: map of Brazil, with Minas Gerais state (MG) highlighted; b: Highlighted in light grey, the SCNP.

Examined specimens (n = 31) were all collected within SCNP (Fig 1), in the headsprings of São Francisco River (-20.2442, -46.4468): 18♀ *F*. *franciscoi*, 13♀ *F*. *sonia*. Surveys were conducted in May 2017, October 2017, March 2018, November 2018 and April 2019. All specimens were collected in accordance with Brazilian law under a scientific collection license (SISBIO license 54386–6). The specimens are deposited at Laboratory of Ecological Studies on Ethology and Evolution (LESTES), UFSCar, São Carlos, Brazil. Animal handling was carried out in strict accordance with the normative instruction of the Instituto Chico Mendes de Conservação da Biodiversidade (ICMBio).

Regarding taxonomy, the specimens were identified upon consulting the description paper [1] and the nomenclature used throughout this study follows [1] and [16] for body morphology. All measurements are in mm. Abbreviations for structures used in the text are as follows: S1–10: abdominal segments 1 to 10.

### Color pattern assessment

Specimen photographs were taken with a Canon Eos 5D Mark II digital camera, Tamron 60mm macro lenses and a Magic Elf Led Macro Flash ML-3D. Dissection images were captured under an Olympus SZ60 binocular microscope with a Leica camera MC170 HD. Images were posteriorly cropped and their backgrounds removed using Adobe Photoshop® without altering any colors, brightness or exposure. After obtaining the images, we visually checked body coloration for both species, searching for similarities and coloration marks that may result from ontogenetic color changes on the head, thorax (dorsal, lateral and ventral) and abdominal segments 1−2 and 8−10; these being areas where both species exhibit colored spots and other species have shown to present such differences [8,12].

### Morphological assessment

To check for potential morphological differences, we examined the traits of both females. In the description of *Franciscobasis* females by [1], the prothorax, mainly the posterior lobe, of both females presents subtle differences. Thus, we made SEM images of these structures to evaluate the degree of resemblance between the prothorax females of *F*. *sonia* and *F*. *franciscoi*. Prothoracic SEM images were taken in CACTI (Univ. de Vigo) with a Phillips XL-30.

To determine female egg maturation, we dissected the abdominal region (n = 10, 5♀ *F*. *franciscoi* and 5♀ *F*. *sonia*). We dissected females preserved in absolute ethanol for at least ten months. The tissues of such females are easier to manipulate, as they are less elastic when compared to recently collected females (Adolfo Cordero-Rivera and Olalla Lorenzo-Carballa pers. comm.). We pinned the females on a small dish covered with a layer of opaque silicone to facilitate the dissection process and filled the recipient with distilled water to prevent the tissues from becoming brittle. Using a pair of fine-tip dissecting forceps, we opened the abdomen from the dorsal tergites, following the medial suture, which is easier to cut. Once the exoskeleton was cut, we opened the abdomen with the aid of small pins and secured them on the silicon to keep the structures visible. We then proceeded to remove the eggs (when present), removing the full egg masses and the digestive tract below S9 and S10 to ensure we examined the full extension of the ovaries. We followed this nomenclature for the eggs: Mature eggs are usually oval, present a resilient chorion and are hard to break; undeveloped eggs are smaller, with no evident chorion and mostly opaque [17–19]. Dissected females are deposited at Escola de Enxeñaría Forestal (ECOEVO), Universidade de Vigo, Pontevedra, Spain.

### Genetic analysis

To determine species proximity, the entire genomic DNA of eight specimens from both *F*. *franciscoi* and *F*. *sonia* (Table 1) was extracted using the QIAGEN DNeasy Blood and Tissue Kit (QIAGEN). PCR amplifications were done for part of the mitochondrial Cox1 gene (COI) using the primers OdoF1_t1 (5'- TGT AAA ACG ACG GCC AGT ATT CAA CHA ATC ATA ARG ATA TTG G -3') and OdoR1_t1 (5'- CAG GAA ACA GCT ATG ACT AAA CTT CTG GAT GYC CRA ARA AYC A -3’) of CCDB [20]. PCR conditions for amplification consisted of 1× buffer, dNTP at 0.2 mM, each primer at 0.2 μM, MgCl_2_ at 2mM, 1U Taq polymerase and 2 μl of template DNA, in a total reaction volume of 25 μl. The PCR cycling program used was: 94°C for 2 min, followed by 5 cycles of 94°C for 30 s, 45°C for 40 s, and 72°C for 1 min, followed by 35 cycles of 94°C for 30 s, 51°C for 40 s, and 72°C for 1 min and concluding with a 10 min extension at 72°C. Sequencing was performed using the M13F (5'- TGT AAA ACG ACG GCC -3') and M13R (5'-CAG GAA ACA GCT ATG AC -3') primers on an ABI 3500 Genetic Analyzer (Applied Biosystems).

**Table 1:** List of sequences from GenBank used in this study (COI). ♂: male specimens; ♀: female specimens.

GenBank accession number	Species		Reference
MK598039	*Franciscobasis*	*franciscoi* ♂	This study
MK598036	*Franciscobasis*	*franciscoi* ♂	This study
MK598035	*Franciscobasis*	*franciscoi* ♂	This study
MK598037	*Franciscobasis*	*franciscoi* ♀	This study
MK598041	*Franciscobasis*	*franciscoi* ♀	This study
MK598038	*Franciscobasis*	*sonia* ♀	This study
MK598040	*Franciscobasis*	*sonia* ♀	This study
MK598034	*Franciscobasis*	*sonia* ♀	This study
KF369273	*Acanthagrion*	*phallicorne*	[21]
KY947441	*Acanthagrion*	*aepiolum*	[22]
KY947440	*Acanthagrion*	*aepiolum*	[22]
KY947446	*Acanthagrion*	*cuyabae*	[22]
KY947447	*Acanthagrion*	*cuyabae*	[22]
KY947437	*Acanthagrion*	*cuyabae*	[22]
KY947438	*Acanthagrion*	*cuyabae*	[22]
KY947439	*Acanthagrion*	*cuyabae*	[22]
KY947449	*Aeolagrion*	*dorsale*	[22]
KY947450	*Aeolagrion*	*dorsale*	[22]
KY947448	*Argentagrion*	*ambiguum*	[22]
KY947427	*Argia*	*smithiana*	[22]
MK598042	*Franciscagrion*	*franciscoi*	This study
KY947410	*Homeoura*	*nepos*	[22]
KY947411	*Homeoura*	*nepos*	[22]
KY947412	*Homeoura*	*nepos*	[22]
KY947467	*Oxyagrion*	*terminale*	[22]
KY947399	*Oxyagrion*	*sulmatogrossense*	[22]
KM536053	*Ischnura*	*verticalis*	Unpublished
KM535109	*Amphiagrion*	*abbreviatum*	Unpublished
KM534075	*Amphiagrion*	*abbreviatum*	Unpublished
JF839353	*Amphiagrion*	*abbreviatum*	Unpublished
KF257113	*Ischnura*	*asiatica*	[23]
KT957493	*Ischnura*	*aurora*	Unpublished
KT708070	*Ischnura*	*kellicotti*	[24]
GQ256032	*Ischnura*	*elegans*	[25]
KF257118	*Ischnura*	*elegans*	[24]
KT879905	*Ischnura*	*senegalensis*	Unpublished

We used GENEIOUS v 7.1.3 [26] to check sequence quality of both strands in comparison to their respective chromatograms, and to assemble and edit if necessary. Our DNA sequences were compared to and evaluated with COI fragments of closely related species obtained from GenBank. These species were chosen through the morphological proximity and previously published molecular phylogenies [21,22,27]. For an overview of all samples and GenBank accession numbers see Table 1. Furthermore, we aligned sequences using Muscle v3.8.425 ([28], module implemented in GENEIOUS v 7.1.3) with default settings.

The genetic distances between and within species were estimated using the Kimura-2-Parameter (K2P) distance model (but see [29]) using MEGA v 7.0.26 software [30]. In order to increase robustness in the homology statement and elevate matrix occupancy, long sequences were truncated to cover only the ‘Folmer’ region of the COI gene; this is the most commonly used region for DNA barcoding, and covers 658 nt of the 5′-end of the gene. For insects, the region can be amplified using the ‘Folmer’ primer pair (HCO2198 and LCO1490), and truncation was carried out following the positioning of these primers (see [31,32]).

Phylogenetic analysis was performed using Maximum Likelihood (ML) in MEGA v 7.0.26. The bootstrap consensus tree was inferred from 1,000 replicates taken to represent the evolutionary history of the taxa analyzed. Finally, we assessed the taxonomic status of *Franciscobasis* species using three species delimitation methods, Automatic Barcode Gap Discovery (ABGD) [33], the Poisson tree processes (PTP) and Bayesian implementation of the Poisson Tree Process (bPTP) [34]. We performed ABGD analyses online (http://www.abi.snv.jussieu.fr/public/abgd/), using K2P model with parameters set to default values, except for relative gap width (0.5). For PTP and bPTP methods, a tree was constructed with RAxML (v 8.2.12) [35] with GTRCAT model and 500 bootstrap replicates, and the analyses were conducted in the PTP web servers (http://species.h-its.org/) using default settings. Resulting species delimitations are presented with the phylogenetic tree.

## Results

### Color pattern assessment

**Fig 2:**
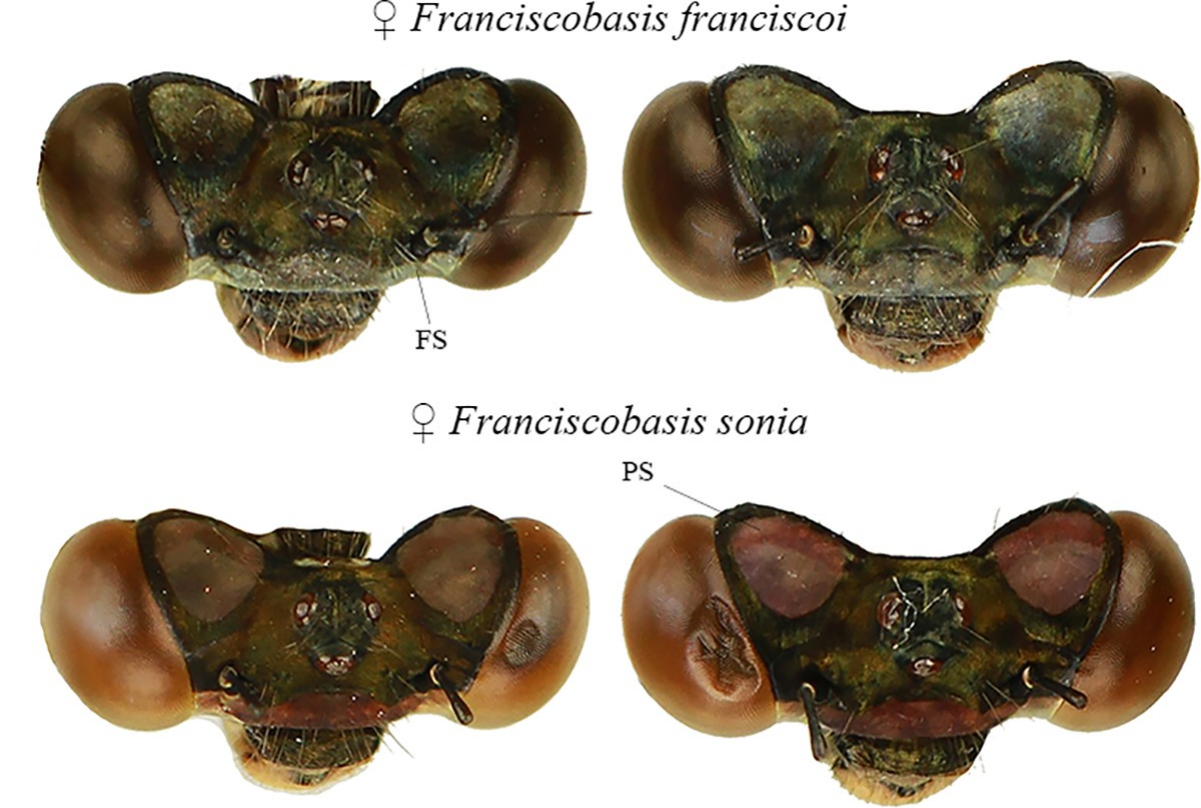
Coloration similarities between *Franciscobasis sonia* and *F*. *franciscoi*. FS: longitudinal frons stripe; PS: postocular spots.

We found several similarities in body coloration within the two species. For instance, on the head, the shape and size of postocular spots (PS) are very similar. Also similar in shape and size are the longitudinal stripe on the frons (FS), present on both species (Fig 2).

**Fig 3:**
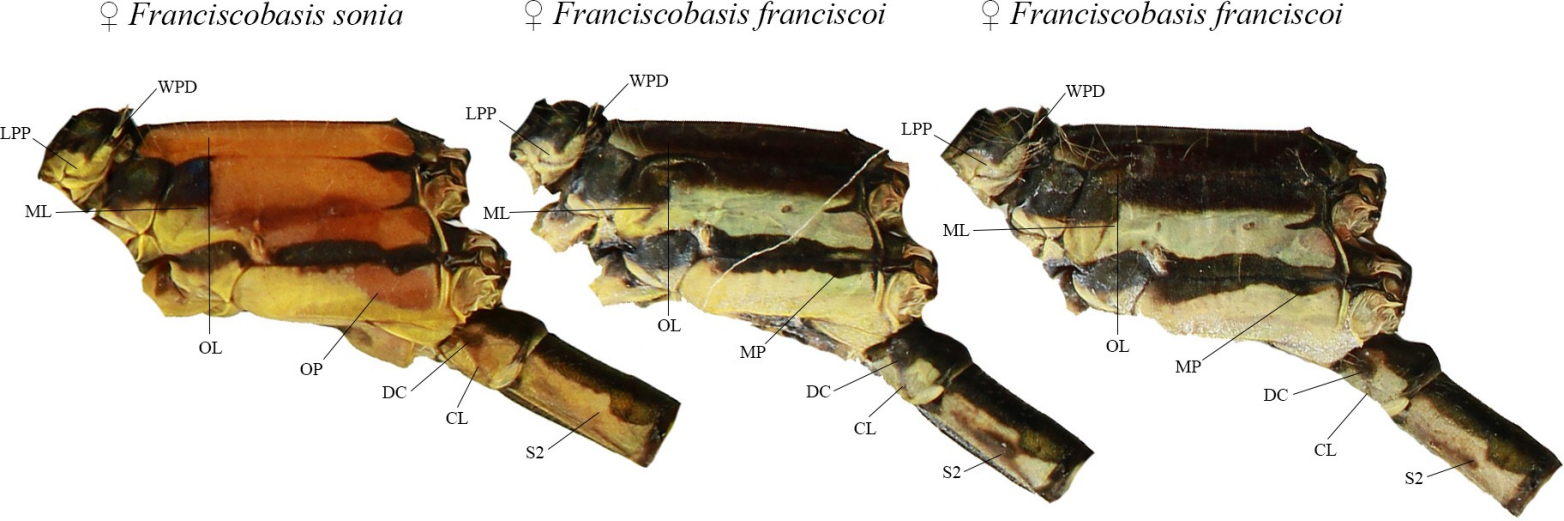
Coloration similarities between *Franciscobasis sonia* and *F*. *franciscoi*. WPD: White posterior lobe dot; LPP: lateral prothoracic pattern; ML: Mesepisternum line; OL: Orange vertical limit of mesepimeron and metepisternum; OP: Orange metepimeron patch only found in *F*. *sonia*; MP: metepimeron black stripe; DC: Dark circle on segment 1; CL: Circle oblique dark line; S2: Color pattern of segment 2.

The lateral thoracic pattern exhibited at least four areas of identical contours. On the prothorax, dots on posterior lobes (WPD) and lateral coloration patterns (LPP) were also identical. Furthermore, the two first segments presented three additional identical color patterns (Fig 3). The orange vertical limit (OL) that comprises the mesepimeron, and about 1/3 of metepisternum presents one of the major similarities between the two species. In addition, the OL makes an angle of almost 90° with the black/pale limit in mesepisternum and mesinfraepisternum, a pattern observed in all females examined. Only specimens of *F*. *sonia* exhibited the orange patch on the posterior portion of metepimeron (OP). However, both species presented a curved line on the upper right portion of metepimeron (MP), immediately above the OP. Black lateral marks on S1−S2 were also very similar. Two particular regions also showed similarities in coloration on lateral S1: a dark circle (DC) on the left portion and a thin oblique line (CL). On S2, the black dorsal pattern was identical, except for some black patches only present in *F*. *franciscoi* females.

**Fig 4:**
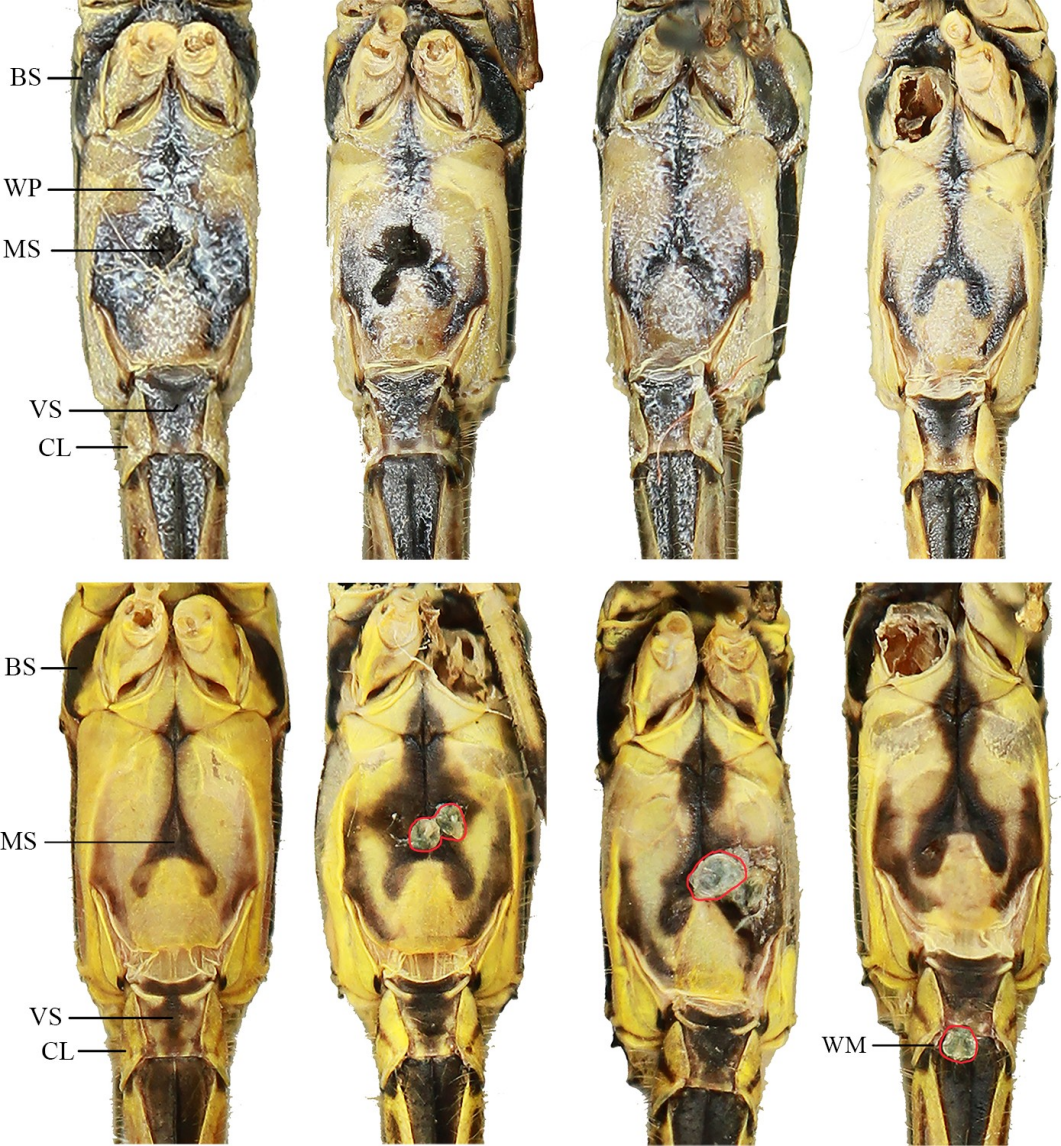
Coloration similarities between *Franciscobasis sonia* (below) and *F*. *franciscoi* (above). BS: Black stripe; WP: White pruinosity; MS: Midventral black stripe; VS: Dark ventral spot; CL: Circle oblique dark line; WM: Water mites.

We also found coloration similarities between the two species in the ventral portion of the thorax and S1−S2 (Fig 4). The main character is the midventral black stripe (MS) which forks at the base of the mound like ventral tubercle. This stripe seems to vary considerably in shape and range between the two species. Another black stripe (BS) that originates at the metepisternum and passes between meso and metacoxae through the ventral thorax is present in both species. A white pruinosity, present only in mature individuals [36,37], occurred only in *F*. *franciscoi* specimens. Although we noticed some variation in the amount of pruinosity, we did not find any *F*. *franciscoi* female lacking pruinose areas. Furthermore, no *F*. *sonia* females presented pruinosity. On ventral S1, a dark spot (VS) is also present in all specimens examined, exhibiting different shapes and sizes, often circular. The dark oblique line was also observed in ventral view (CL).

**Fig 5:**
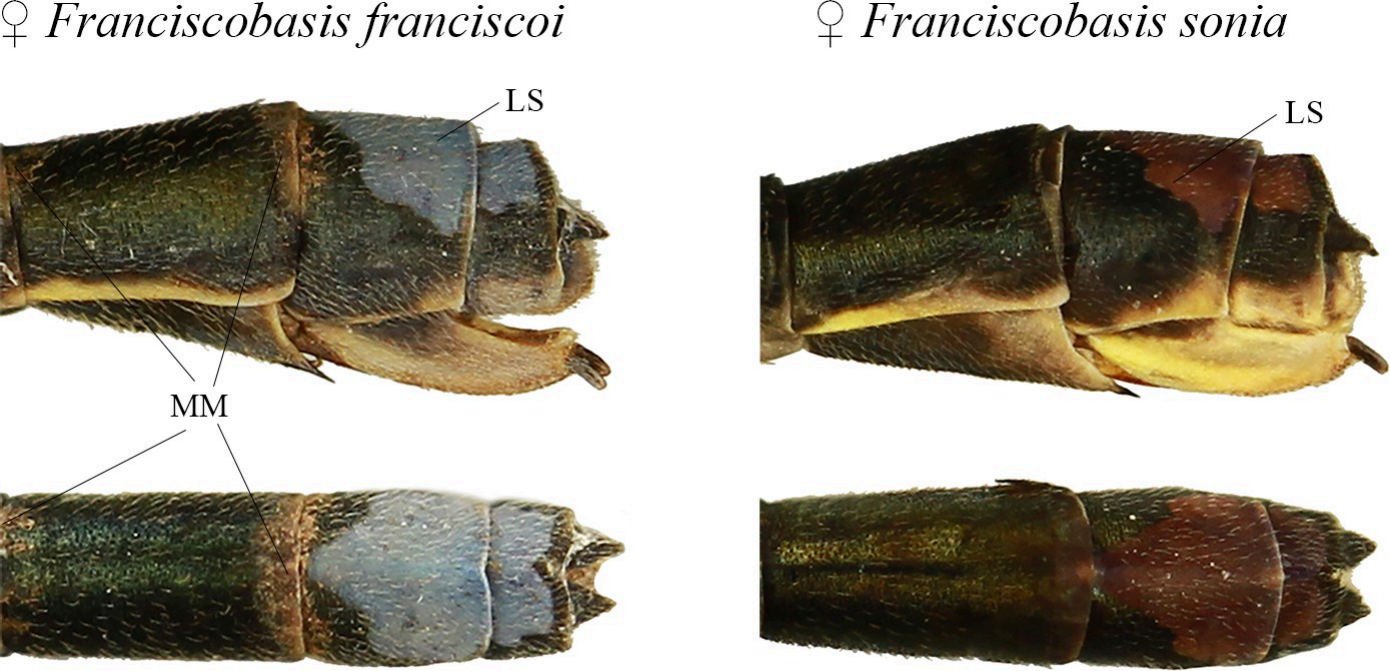
Coloration similarities between *Franciscobasis sonia* and *F*. *franciscoi*. LS: large dorsal spot; MM: Mud marks.

At the end of the abdomen (Fig 5), the large dorsal spot (LS) is very similar in size and shape between the two species, being blue in *F*. *franciscoi* and violet in *F*. *sonia*. Exclusively in *F*. *franciscoi*, most of the captured females presented mud marks on the final abdominal segments.

### Morphological assessment

**Fig 6:**
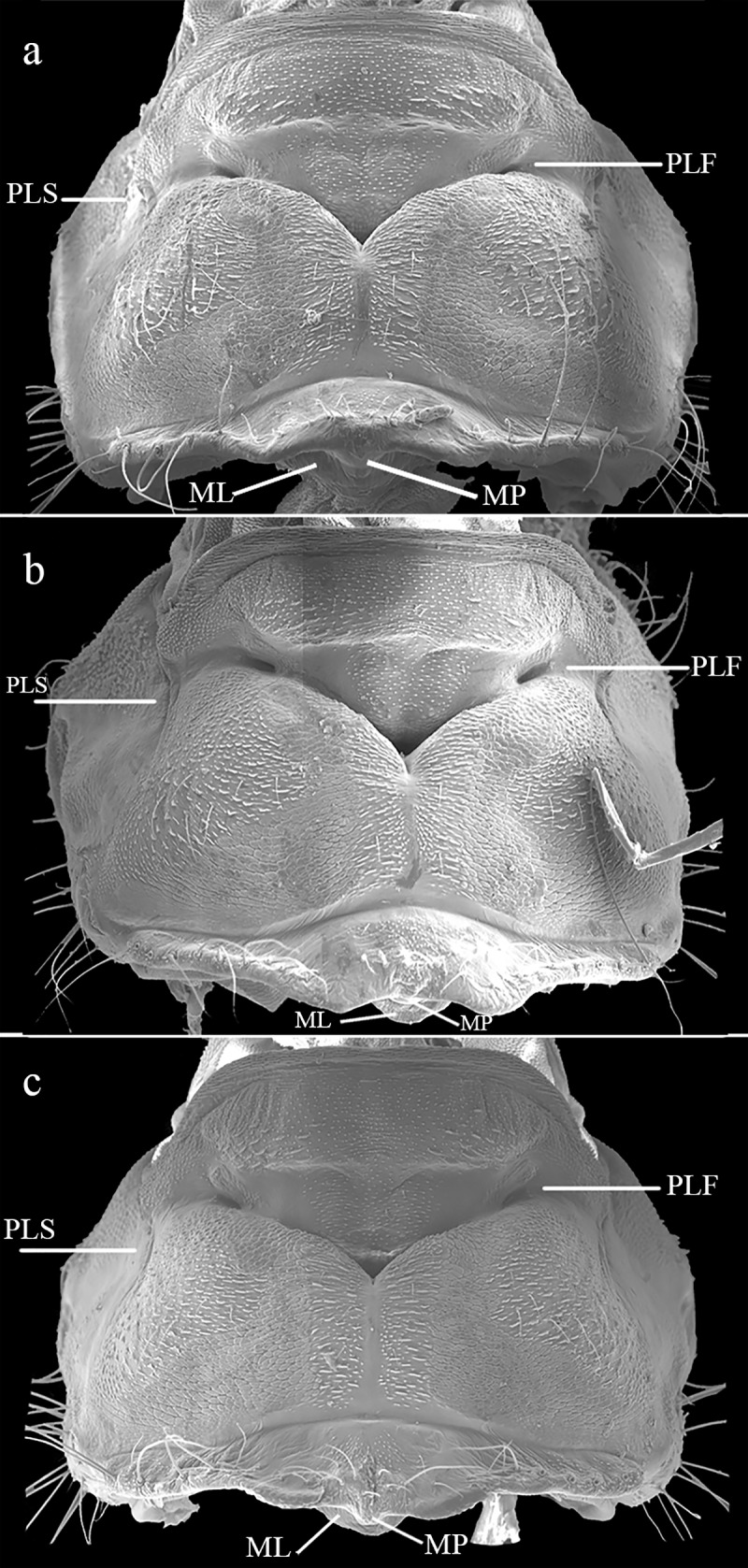
SEM images of dorsal pronotum, showing the main characters present on this structure. 6a–b. *F*. *franciscoi*; 6c. *F*. *sonia*. PLS: notopleural suture; PLF: Lateral fossa; ML: Middle lobe of posterior lobe; MP: Rounded middle projection.

Upon analyzing the morphological structures of prothorax, we found few variations among the examined specimens, which may be due to different angulation on the images. The morphological traits of the posterior lobes were present on all examined females, varying slightly in size. The SEM images allowed us to notice some additional structures, unrecognizable in optical microscopy photos. The prothorax is mainly characterized by two lateral fossae (PLF) between anterior and medial lobe, an identical notopleural suture (PLS), two lateral lobes slightly curved upwards and one middle lobe (ML) bent caudad, with a small rounded projection (MP) above its middle portion (Fig 6A–6C).

The dissections showed a consistent pattern related to egg presence and development. Only females of *F*. *franciscoi* presented mature eggs (E), measuring 327±23 μm (n = 55). The eggs presented a resilient chorion when pressed, and only broke when smashed with the forceps (S1 Fig).

On the other hand, dissected females of *F*. *sonia* only presented an undeveloped egg mass (UEM) with eggs measuring 46±15 μm (n = 11, an estimatation, as the eggs are not clearly separable). This UEM, when present, was closely attached to the digestive tract (DT), was difficult to remove without adjacent structures and presented a soft consistency. In some females (n = 2) the UEM was almost imperceptible, with the DT observable only at the moment of the abdominal opening (S2 Fig).

### Genetic analysis

**Fig 7:**
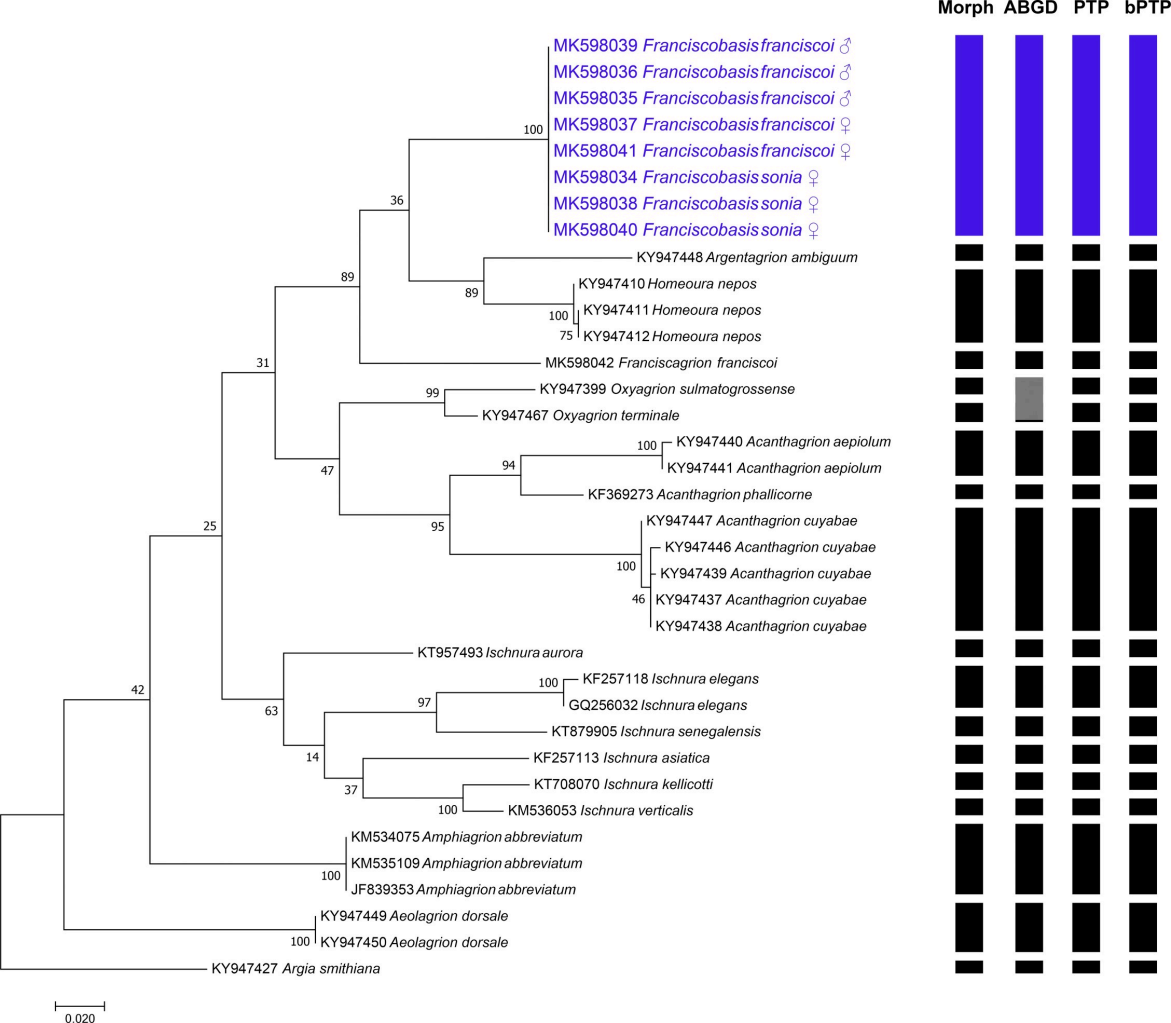
Maximum likelihood phylogenetic tree of *Franciscobasis* species based on the COI gene. Shown on the right are the results of species delimitation analyses using morphological characters (Morph), Automatic Barcode Gap Discovery (ABGD), Poisson tree processes (PTP) and Bayesian implementation of the Poisson Tree Process (bPTP), respectively.

The target COI sequences of all samples amplified were sequenced successfully. The K2P distance of COI sequences among *F*. *franciscoi* and *F*. *sonia* sequences was 0.00% - 0.16%, while for the other species this value ranged between 10.82% and 20.56% (S1 Table). *Franciscobasis* genus formed a monophyletic group with strong support (i.e., 100% bootstrap; Fig 7) and the three species delimitation methods recognized *F*. *franciscoi* and *F*. *sonia* as a unique species.

## Discussion

All our evidence shows that *Franciscobasis sonia* is the same taxon as *F*. *franciscoi*. We were unable to separate them morphologically, relying mainly on the dorsal prothoracic characteristics. Our images are quite different from the original description figures, which may be due to improper position of prothorax at the time of drawing (thus, hiding some diagnostic structures such as ML and MP). In most of the coenagrionid genera, females vary morphologically among species, and the most common structures used to separate them are those from the prothorax, as in *Ischnura* [16,38]. In other genera such as *Cyanallagma* [39] and *Epipleoneura* [40], females can be separated specifically using the morphology of the posterior lobe. *Franciscobasis* females presented an almost identical prothorax structure. Although structures vary slightly in size (even between conspecifics), we were unable to clearly separate them, due to the high resemblance. In comparison, in *Amphiagrion* (a genus that shares a ventral thoracic tubercle and is morphologically close to *Franciscobasis*, see [1]), although female mesostigmal plates are similar, they can easily be separated by the presence of strong tubercles in *A*. *abbreviatum*, which are less prominent in *A*. *saucium* [40]. In *Franciscagrion* and *Homeoura* (two genetically close genera), the prothoracic posterior lobes are diagnostic and differ enough to separate the species [1,38].

Since we found no external differences in morphology of the females, we proceeded with the dissection process. Only *F*. *franciscoi* females presented developed eggs (with a resilient chorion, difficult to break and translucid, *sensu* [17]). On the other hand, *F*. *sonia* females presented an opaque UEM, with small undeveloped eggs. In Odonata, it is possible to assume female age by the number and size of the mature oocytes (e.g. eggs) present in their ovaries. Young females often present a narrow bundle of undeveloped ovarioles placed closely to the intestine, and mature females present hundreds of developed ovarioles [41]. Additionally, we only found muddy marks on *F*. *franciscoi* females, which may indicate oviposition in muddy areas [5]. These patterns lead us to consider that *F*. *sonia* specimens were, in fact, young *F*. *franciscoi* females.

Concerning coloration, despite the great differences between *Franciscobasis sonia* and *F*. *franciscoi*, we found many similarities between both species. First, the postocular spots present the same shape and size, varying only in coloration. In other Coenagrionids which present postocular spots, such as in *Acanthagrion* and *Argia*, females have great interspecific variation in color, shape, and size of these spots [40,41]. Postocular spots can also be highly variable, or even absent in individuals belonging to the same species, like in *Ischnura graellsii* [6]. On prothorax and S1−S2, we found similar marks of coloration on both females. The main evidence of ontogenetic color changes on this area is the orange vertical limit (OL; black in *F*. *franciscoi* females) that makes an angle of almost 90° with the black/pale limit in mesepisternum and mesinfraepisternum: A pattern observed in all females examined from both species (Fig 2). In Coenagrionidae, mainly *Ischnura*, females are known to undergo a complete coloration change from emergence to sexual maturation, being light green, violet or orange as young females, and becoming brownish and black as they grow old [8,9]. Thus, we suggest that this pattern of ontogenetic color change may occur with *Franciscobasis* females as well. Furthermore, pruinescence, a sign of maturity, was found in most of the females of *F*. *franciscoi* and no females of *F*. *sonia*.

Furthermore, the results of the molecular analysis were consistent with morphological and coloration evidence, indicating a genetic similarity of 100% between the examined females. In fact, DNA barcoding has been used to confidently associate aquatic and terrestrial life stages, especially in Odonata [22,42,43]. This technique has previously shown to be effective with larvae of *Drepanosticta attala* Lieftinck 1934 [43] and *Onychargia atrocyana* (Selys 1865)[42]. Therefore, with all specimens examined forming a unique clade, and the almost zero K2P distance of COI sequences, we believe to have further evidence to imply that they must be the same species.

Integrating the findings of different disciplines, such as morphological (external and internal) and molecular analysis is important in maintaining a rigorous species delimitation process and avoiding taxonomic inaccuracies [5]. In the case of our study, only the traditional external morphology assessment would prevent us from concluding the identity of the studied specimens. Although good alpha taxonomy is imperative to biology, an approach that explores different sources and disciplines may assist us in looking further at the integrative approach of species delimitation, and could help to better understand lineage diversification without replacing the traditional view on taxonomy [4,5].

In conclusion, considering our evidence, and with respect to Article 24.2 of the International Code of Zoological Nomenclature, we suggest that *Franciscobasis sonia* is a junior synonym of *F*. *franciscoi*, as the latter was the first one to be described.

## Supporting information

S1 Fig Dissection of a female *Franciscobasis franciscoi*. a. an example of the dissection procedure, when females were opened from the dorsal tergites following the medial suture, to expose the eggs (E); b–c. mass of eggs, removed from the abdominal cavity; d. an egg, separated from the egg mass.
(TIF)

S2 Fig Dissection of a female *Franciscobasis sonia*. a. dissection procedure showing only the digestive tract (DT), as in this female the egg mass was not yet perceptible; b–c. dissected female, showing an undeveloped ovariole and egg mass lying dorsal to the DT (UEM); UEM removed from the abdominal cavity.
(TIF)

S1 Table K2P distances for Coenagrionid species. Aligned sequences of the COI of the *Franciscobasis* species and 17 other coenagrionid species (see Table 1).
(TIF)
