# Supplementary figures and images for: A further study on Franciscobasis Machado & Bedê, 2016 (Odonata: Coenagrionidae), a newly described genus from Minas Gerais, Brazil
Source: PLoS One. 2019 Oct 8;14(10):e0223241. doi: 10.1371/journal.pone.0223241 (PMC6782088; doi:10.1371/journal.pone.0223241)

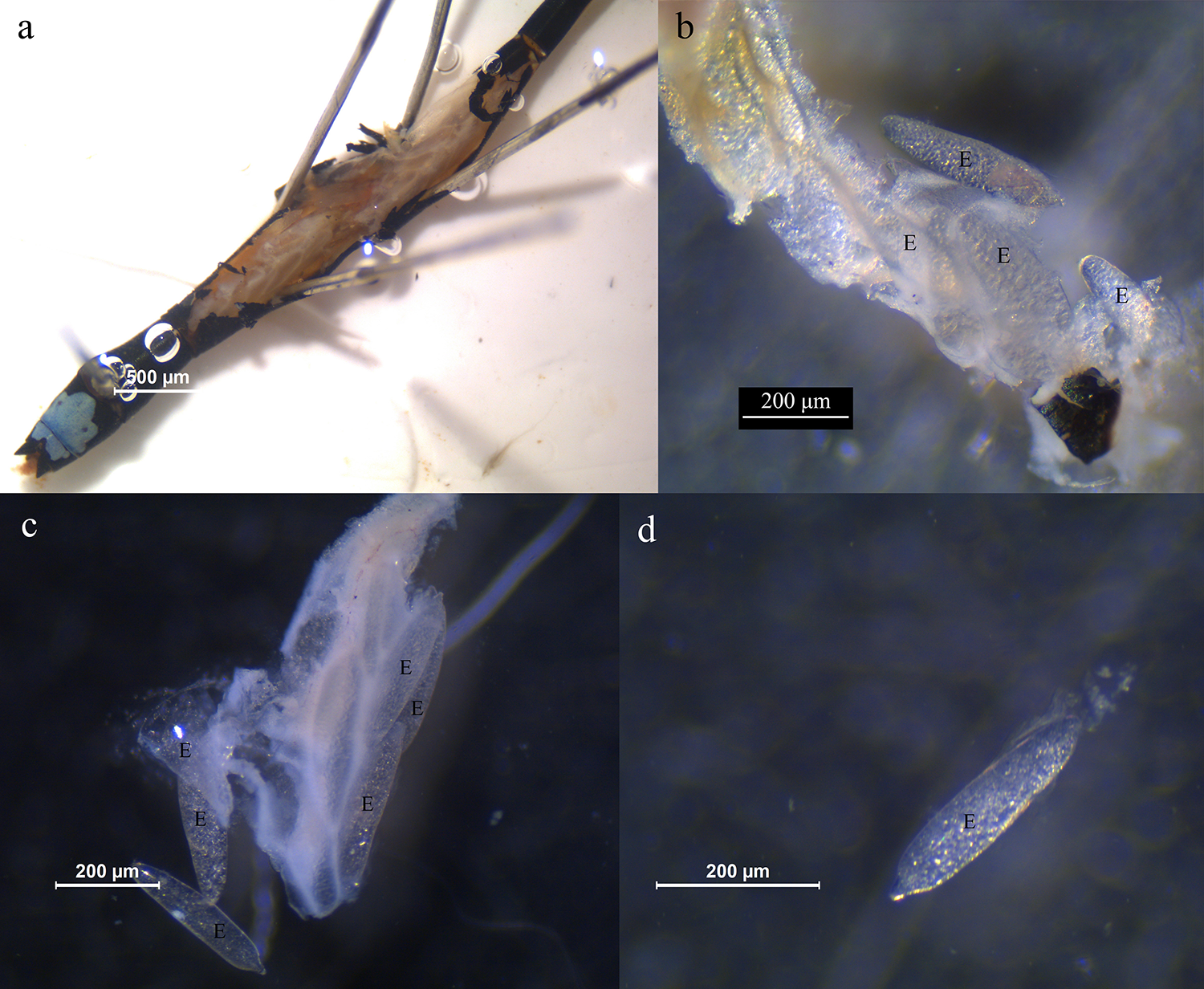

Supplement: S1 Fig — a. an example of the dissection procedure, when females were opened from the dorsal tergites following the medial suture, to expose the eggs (E); b–c. mass of eggs, removed from the abdominal cavity; d. an egg, separated from the egg mass. (TIF) [file pone.0223241.s001.tif]

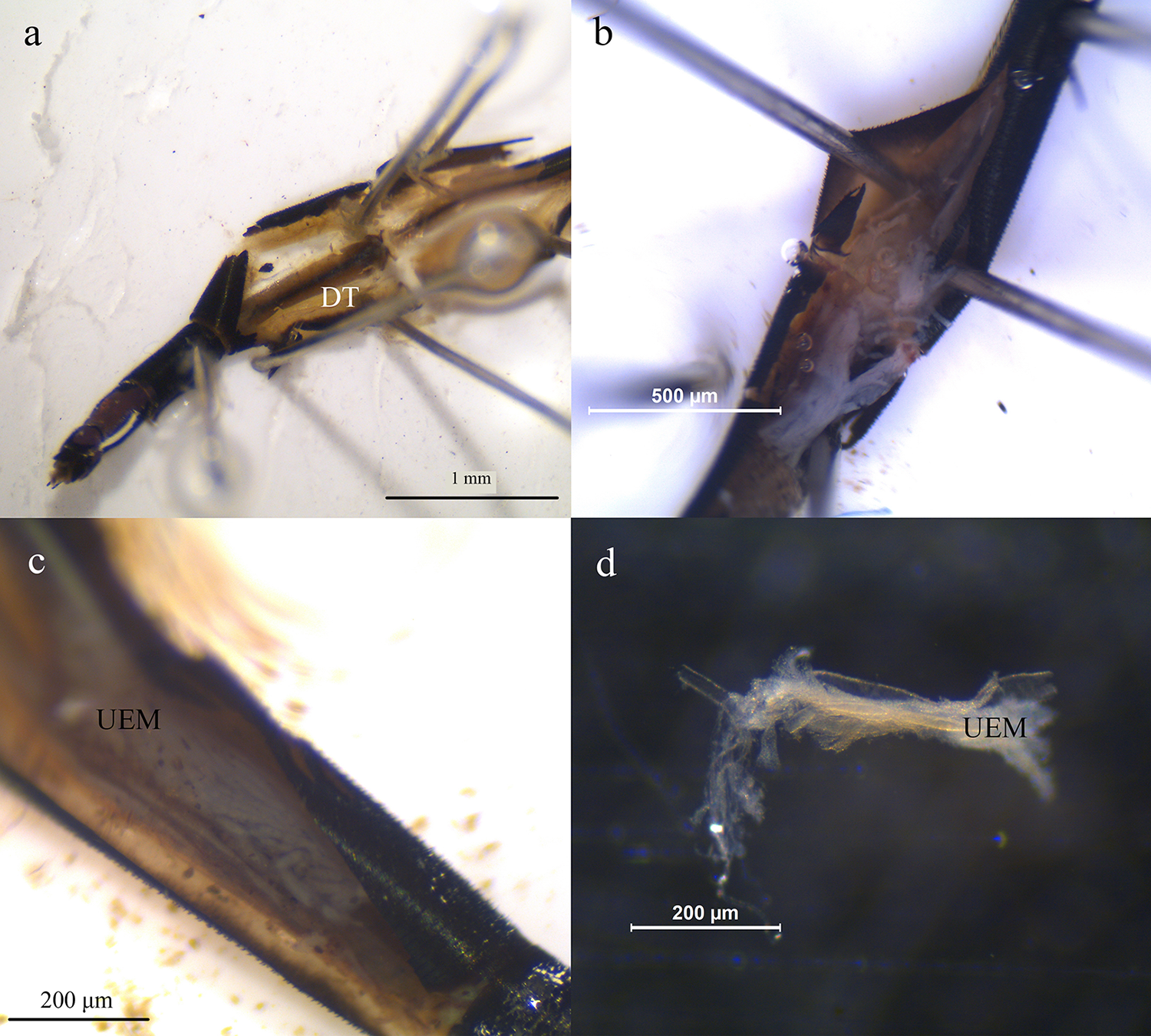

Supplement: S2 Fig — a. dissection procedure showing only the digestive tract (DT), as in this female the egg mass was not yet perceptible; b–c. dissected female, showing an undeveloped ovariole and egg mass lying dorsal to the DT (UEM); UEM removed from the abdominal cavity. (TIF) [file pone.0223241.s002.tif]

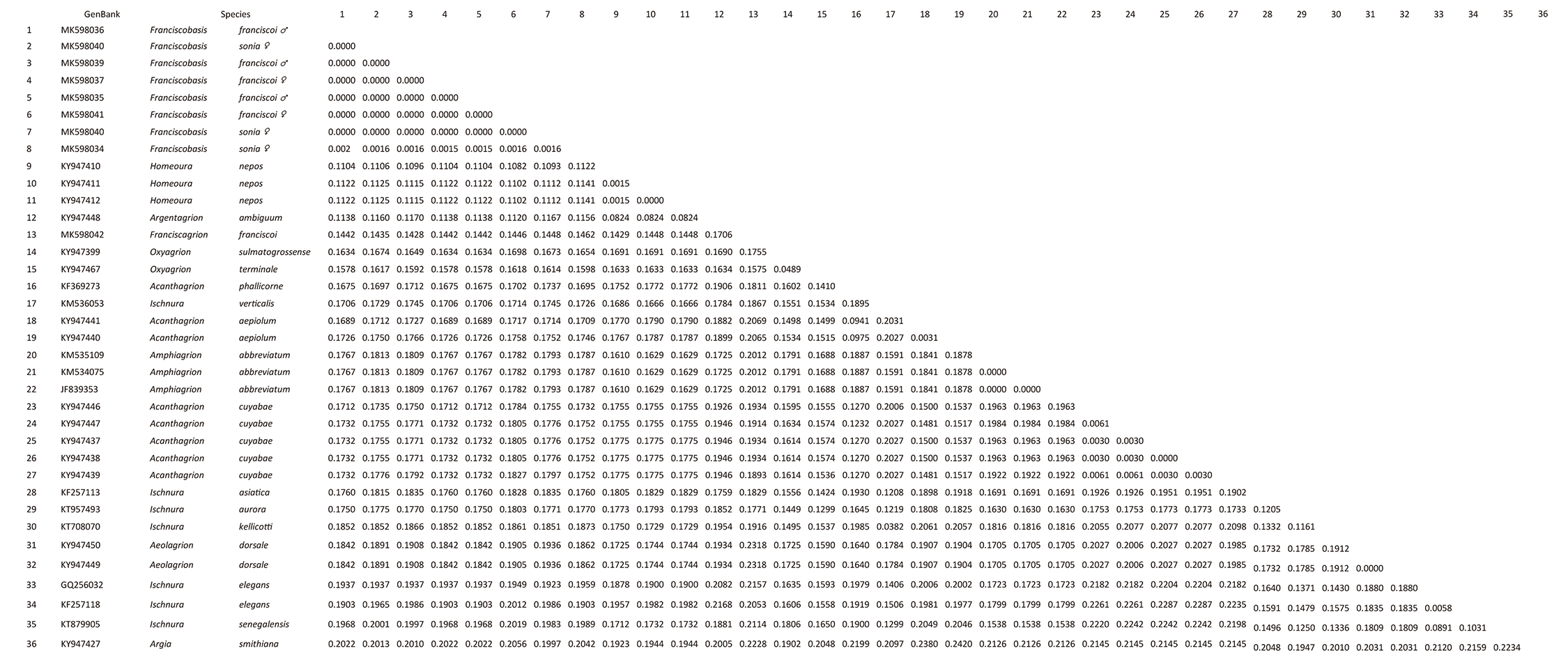

Supplement: S1 Table — Aligned sequences of the COI of the Franciscobasis species and 17 other coenagrionid species (see Table 1). (TIF) [file pone.0223241.s003.tif]
